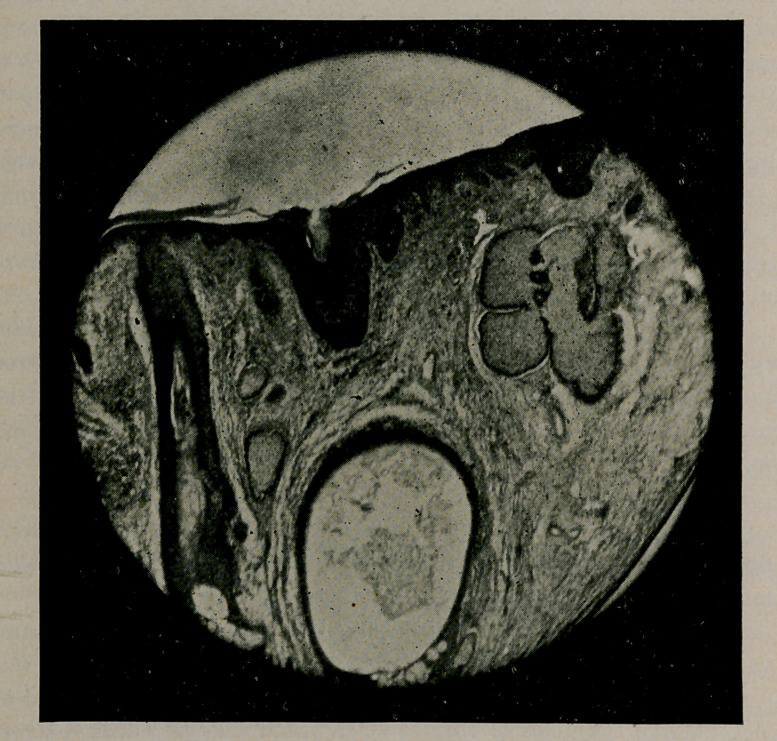# Rhynophyma

**Published:** 1899-10

**Authors:** Ernest Wende, Grover William Wende

**Affiliations:** Professor of dermatology, University of Buffalo; Clinical professor of dermatology, University of Buffalo


WENDE: RHYNOPHYMA.

### Special Article.

### RHYNOPHYMA.

By ERNEST WENDE, M. D.,
Professor of dermatology, University of Buffalo,

AND

GROVER WILLIAM WENDE, M. D.,
Clinical professor of dermatology, University of Buffalo.

THE accompanying illustration conveys an excellent idea of a Bohemian, 48 years of age, with the unequivocal marks of the condition under consideration graphically portrayed. He is a burly looking gentleman, of gaunt and intermediate stature, erect carriage, and buoyant spirits. Barring the disfigurement, which is singularly acute, he possesses fair features with brown hair. His habits, excepting an unquenchable thirst, are good. His fondness for beer is especially well established. It is his custom, during the hours of employment, rather to palliate the morbid desire for drink than to be confused by the uncertainty of taking too much. Productiveness becomes the result of submission. Disposed to joy and pleasure, cares disappear, and, dissatisfied with the drugging of the week, he consecrates Saturday evening and Sunday, by taking a full course, to enjoy a relaxation suitable to his means and peculiarities. His bibulous propensities were contracted early in life, and have continued to the present time, yet, in defiance of this manner of living, he was always industrious and never indolent.

The unwisdom of these beery indulgences at fixed periods often induced intestinal distress and diarrhea, which were additional penalties, in his case, for a violation of nature's laws. Nature is relentless, and knows nothing of extenuating circumstances; she never forgets and never forgives.

The digestive impairment brought about by his rhythmical habits of an unmitigated curse, that exercised such an injurious influence upon the significance of his nose as an attribute of beauty, is a just recognition of the futility of fighting against nature.

It is not a pleasant picture, by any means, but it happens to be a truthful portraiture, and it will not mend matters to close our eyes against the facts. Such facts should be an effectual check upon the vast majority of those who might wish to imitate. The effects, like the beverages, may be mixed and mingled in all proportions, as an equitable adjustment to suit the taste of the consumer.

It would be idle for the physician, in a disease like this, where there is so much to admire that is interesting and complex in its properties of organised structure, to excite a morbid repugnance. He will find it more profitable to carefully study the circumstances which interrupted the harmonious equilibrium of the various functions performed by the skin, with a view of developing an appreciation of its pathological departure, character and gradations.

It is manifestly easy to divide the evolution of acne rosacea into three stages, merging into one another of which rhynophyma, consisting of a gradual hypertrophy of elastic tissue, often of monstrous proportions, represents the final termination. The first indication of embarrassment to our patient was a diffuse redness, particularly confined to the extremity of the nose which was disturbed on slightest provocation, becoming more vivid in appearance when exposed to sudden vicissitudes of temperature, or after a hearty repast, and especially when associated with the glass that cheers. For a long time, following the inception of the disease, the erubescence was localised and transitory in character, but after a while it diffused itself over the entire nose, and finally extended in a symmetrical manner over the contiguous surface, involving the greater portion of the cheeks, forehead and chin. The discoloration now became permanent and more profound.

The capillaries were dilated and tortuous and the blood circulated slowly through them. There was a tendency toward stasis. The condition of the skin was decidedly unctuous and cold to touch, yet, at intervals, a sensation of heat and fulness prevailed, depending undoubtedly, upon some special source. The hyperemia, which was at first recurrent and then permanent with exacerbations, soon developed papules in the papillary layer, and likewise as a complication, acne pustules. This state of affairs, since its incipiency, has been about twenty years in formation, when the patient, participating in a melee, was struck by a brick, causing three distinct ruptures of the skin, radiating from the tip of the nose. The necessary stitches were promptly taken, the wound properly dressed which healed quickly with a marvelously good result. However, shortly thereafter, it was noticeable that the organ began to enlarge with perceptible rapidity for two years, and then gradually to reach its present proportions.

The hypertrophy at first was especially well-marked at the point of injury. The acne papules and pustules, which from time to time were found disseminated over the hyperemic area, have entirely disappeared. They were practically nonexistent one year ago.

After this cursory consideration, it is discernible that the persistent hyperemia caused an induration of the skin, the glands and bloodvessels of the whole nose, while dilated venules course over its surface. The hypertrophic changes which have ensued to produce this degree of distortion in our subject, assume the appearance of an embossment clump or bump that can be resolved into divers conspicuous and well-defined masses or lobes, separated by deep clefts and wide fissures. These rifts, which form the capital letter H at the summit of the nose, are particularly well pronounced, splitting it into four irregular rough and tubercular eminences. The columella is but slightly involved, while the end and the sides of the nose are greatly expanded, by a persistent, intensely florid, but non-inflammatory nodulated thickening which, upon palpation, is doughy and elastic. The skin is coarse, uneven, and studded with numerous deposits of a hard inspissated sebaceous matter, and its surface is pitted with a profusion of pores, some of which gape widely and, upon pressure, a vast quantity of sebum, emitting a disagreeable rancid odor, can be liberated. Concerning the color, it may be said that in the beginning it was a gorgeous red which, up to a certain period, as time went on, mysteriously and unperceptibly changed into a Bishop's purple, but, with advancing age, again began to fade so that now it has assumed almost a normal tint.

This case is instructive, in that the history reveals, in a satisfactory manner, all the characteristic features that enter into the three stages that go to make up this chronic composite affection of the face and particularly of the nose, which is generally years in formation.

To particularise in brief:

1st stage.—A recurrent hyperemia, in the form of an erythematous redness, which occurs in irregularly-shaped patches, giving the skin a mottled aspect accompanied with an inclination to stasis.

2d stage.—A permanent hyperemia often associated with acne and a varicose state of the various radicles of the skin.

3d stage.—A hypertrophy of fibrous tissue in the form of red or violet colored tubercles of various size, which may be either discrete or confluent.

Predisposition, like in many other skin manifestations, is a necessary factor, but the exciting causes are, for the most part, different in the two sexes. In women, the condition of the system that precedes a menstrual period is a universally recognised element. In men, according to the views of numerous authorities, it is an over-indulgence in alcohol.

In this instance, too, the beer was grateful to our patient at the time, whatever the after-effects. Yet, we are of the opinion that, in this case, the injury had much to do in determining rhynophyma, the third and highest degree of the malady which is quite rare, while the first two stages, like "nipping," are common. Then, again, it has occurred in persons of temperate, even of abstemious habits.

The suppletory photo-micrograph, emphasises the pathological evidences most strikingly. The epidermis, maintaining its natural outline, is considerably thinner than normal, and the interpapillary processes extend much deeper into the underlying corneum. The bloodvessels are increased in caliber, and their coats much thickened.

The sebaceous glands are greatly enlarged and filled with sebum, while their ducts, with somewhat hypertrophied walls, terminate in a funnel-shaped opening upon the surface.

The large cavity in the illustration defines the presence of a retention cyst lined with a membrane composed of a dense connective tissue, on the interior of which, in some instances, cellular elements may be seen to cling. The connective tissue of the corium is greatly augumented, and it is this which principally gives rise to the hypertrophy and grotesque appearance.

The adaptation of proper therapeutic measures must naturally vary with the cause and stage involved.

After a careful inquiry into all the circumstances in the case under consideration, it was absolutely necessary, early in the disease, in justice to himself and to the public, that he should have submitted gracefully to a regulation of diet and a forbearance of the Circean cup. A will or a determination, on his part, could have prevented veiled insinuations, and avoided a publicity of his wares. However, a great deal can yet be accomplished by judicious interference. The method that promises the most for its ablation would be an excision of the exuberant growths.

In our judgment, the opportunity for its removal by means of galvanism, punctures, scarifications or cauterisation is past.

Beer, honest beer, to what base uses may you not be brought?

Now, he is only at ease when he tempers his sorrow by singing "Auld Lang Syne."

---

Latent Rheumatic Conditions.—The physician is frequently called upon to treat patients, who though not ill enough to be in bed are not at all well. Their appetite is capricious, they sleep indifferently, or even if they sleep soundly they are not refreshed; in the morning they are more fatigued and ill at ease than was the case on retiring. Upon awaking there is frequently an aching sensation in the loins, sometimes in the lower limbs, which is noticed upon getting out of bed or in dressing, particularly in putting on their hose or in lacing their shoes. As the day progresses this soreness may partially wear off, but there is at all times a vague, undefined, uneasy, painful feeling.

A competent examination of the urine in these cases will in almost every instance be found to disclose a notable absence of the soluble urates. On the contrary, it may be loaded with the phosphates and very frequently bile will be present as also uric acid. If the condition remains neglected, the probable results will be sooner or later a pronounced attack of rheumatism in one or another of its forms. All that is needed to induce such a condition is a sudden change in the weather or exposure on the part of the patient to cold or wet, or a combination of the two. This is due to a latent rheumatic diathesis, to which every adult is liable.

In such cases the physician will find tongaline in any one of its forms, as indicated, given at short intervals with copious draughts of hot water, a remedy which goes directly to the source of the trouble. Tongaline seeks out the retained excretions or perverted secretions, which it either neutralises or renders amenable to the physiological action of the emunctories, and then it brings to bear its strong eliminating powers, correcting the complaint promptly and thoroughly. —*Clinical Notes.*

## Figures and Tables

**Figure f1:**
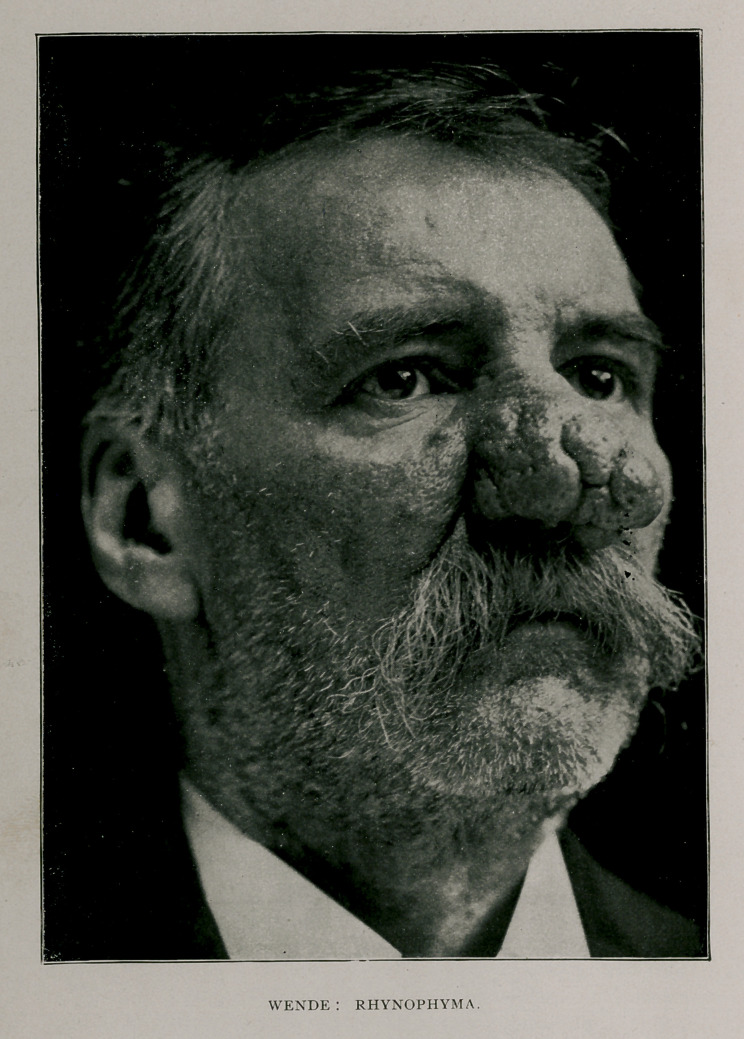


**Figure f2:**